# Beating the odds: medicines alone will not stop HIV

**DOI:** 10.1002/jia2.26321

**Published:** 2024-06-25

**Authors:** Beatriz Grinsztejn, Cristina Mussini, Claudia Cortes, Darrell H. S. Tan, Nittaya Phanuphak

**Affiliations:** ^1^ Instituto Nacional de Infectologia Evandro Chagas‐FIOCRUZ Rio de Janeiro Brazil; ^2^ Università degli Studi di Modena Modena Italy; ^3^ HIV/AIDS Workgroup Faculty of Medicine University of Chile Santiago Chile; ^4^ Department of Internal Medicine Faculty of Medicine University of Chile Santiago Chile; ^5^ Hospital Clínico San Borja Arriarán & Fundación Arriarán Santiago Chile; ^6^ Millenium Institute on Immunology and Immunotherapy Santiago Chile; ^7^ Division of Infectious Diseases St. Michael's Hospital Toronto Ontario Canada; ^8^ MAP Centre for Urban Health Solutions St. Michael's Hospital Toronto Ontario Canada; ^9^ Department of Medicine University of Toronto Toronto Ontario Canada; ^10^ Institute of Medical Science University of Toronto Toronto Ontario Canada; ^11^ Institute of Health Policy, Management and Evaluation Dalla Lana School of Public Health Toronto Ontario Canada; ^12^ Institute of HIV Research and Innovation Bangkok Thailand

1

In the past 20 years, the world has made significant medical progress in addressing HIV. Groundbreaking HIV treatment and prevention options, such as pre‐exposure prophylaxis (PrEP), are increasingly used around the world. As of 2023, 29.8 million of the 39 million people living with HIV (PLWH) globally were receiving HIV treatment [1]. Access to PrEP has increased over 1000% from 2019 to 2022. This increased use of treatment and prevention options has resulted in an almost 60% reduction in new HIV acquisitions in children in 2022 compared to 2010, the lowest since the 1980s, and in almost three‐quarters of PLWH in 2022 having suppressed plasma viraemia; though eastern Europe, central Asia and the Middle East and North Africa have reported increases in new HIV acquisitions.

Indeed, while the global data are encouraging, progress for key populations (KPs)—gay and other men who have sex with men (MSM), sex workers, transgender people and people who inject drugs (PWID) and migrants—is particularly uneven despite their increased vulnerability to HIV. In 2023, outside of sub‐Saharan Africa, the majority of new HIV acquisitions were among KPs [1]. PrEP coverage among KPs in low‐ and middle‐income countries is typically under 5% [1]. Antiretroviral therapy coverage and retention in care are lower for sex workers, transgender people and PWID compared to the general population [1].

Medicines alone will not close this gap. KPs need enabling legal and policy environments to support their access to and uptake of HIV‐ and other health‐related services. In discriminatory and punitive legal and policy environments, KPs avoid HIV‐related services for fear of harassment, discrimination or reporting to law enforcement by healthcare workers. In Argentina, a study found that transgender people who experienced stigma in healthcare settings were three times more likely to avoid seeking healthcare than those who had not experienced stigma [2].

The consequences of a punitive and discriminatory legal and policy environment on KPs and their health are staggering. MSM in countries that criminalize same‐sex relations are more than twice as likely to be living with HIV compared to those in countries without such criminal penalties [3]. A study conducted in 10 countries in sub‐Saharan Africa found that HIV prevalence among sex workers was 7.17 times higher in countries where sex work was criminalized compared to countries where it was not criminalized [4]. A 2017 systematic review found that 80% of included studies reported that criminalization of drug possession had a negative impact on PWID's access to HIV prevention, treatment, care and support services [5].

Yet, no country in the world has repealed laws criminalizing all KP behaviours, including laws related to sex work, possession of small amounts of drugs for personal use, same‐sex sexual behaviour and HIV transmission, exposure or non‐disclosure, despite a global commitment to having less than 10% of countries having punitive legal and policy environments that deny or limit access to services [6].

Indeed, a spate of countries in the last decade has enacted increasingly punitive and discriminatory laws undermining KPs’ access to services [1]. In Nigeria, the enactment of the Same‐Sex Marriage (Prohibition) Act in 2014 resulted in a spike in MSM avoiding healthcare services for fear of being reported to law enforcement and violence against the lesbian, gay, bisexual and transgender (LGBT) community [7]. Similar outcomes were reported in Uganda after the enactment of the Anti‐Homosexuality Act in 2023 [8]. Populist governments in industrialized countries have also been eroding protections for KPs [9].

The restrictions created by these punitive laws and policies combined with growing anti‐democratic sentiment globally is making legal and policy reform increasingly challenging. These laws have heightened security risks for KPs who are often at the forefront of legal and policy reform efforts and thus become targets of government harassment for their advocacy. In Kyrgyzstan, a law banning the dissemination of information on LGBTQ rights was accompanied by other efforts to restrict civic space, similar to efforts in Russia, Poland and Hungary [10].

This heightened risk facing KPs highlights the need for other stakeholders to step up and support legal and policy reform efforts. To do this, we must first ensure that healthcare laws and policies draw a clear line between healthcare and law enforcement, ensuring that healthcare workers and facility and community‐based researchers are not asked or required to provide information to law enforcement on a patient's sexual orientation, gender identity, migrant status, or involvement in sex work or use of drugs. All healthcare workers and researchers should be trained on this practice, and it should be widely understood that any breaches of this policy would result in sanctions.

Second, all HIV and health programming must incorporate legal and policy reform [11]. For instance, country coordination mechanisms addressing HIV and health should intentionally include not just health experts and officials from ministries of health, but also legal and policy experts and officials from ministries of justice and law enforcement. Public health workers and researchers need to work with legal and policy experts in the design, implementation and evaluation of HIV programming efforts.

Finally, a greater and linked portion of HIV funding needs to be allocated for legal and policy reform efforts. In 2022, only about 5% of total HIV resources available were spent on programmes addressing human rights; policy dialogue; reduction of stigma, discrimination and gender‐based violence; and HIV‐related legal services [1]. Further, HIV funding needs to expand to those beyond the government health sector and service delivery‐focused civil society organizations to allied civil society organizations working on law and policy reform.

Solely focusing on medical interventions will not be enough to end AIDS by 2030. Prevention and treatment responses must be optimized by and through legal and policy reform as part of what we do. Further, we must support KP‐led efforts to reform the legal and policy environment through ensuring a clear delineation between our roles as healthcare providers and advocates and supporting law reform. The book AIDS Doctors: Voices from the Epidemic: An Oral History highlighted the crucial role that medical doctors played in the face of uncertainty and death [12]. Similarly, we must now add our voices and partner with those engaged in legal and policy reform efforts to ensure that access to services is a reality for all KPs.

## COMPETING INTERESTS

BG declares no competing interests.

## AUTHORS’ CONTRIBUTIONS

BG wrote the first draft of the report. All authors commented on the report and approved the final version.

## FUNDING

BG is funded by the National Council of Technological and Scientific Development (CNPq) and Carlos Chagas Filho Foundation for Research Support in the State of Rio de Janeiro (FAPERJ).
